# Fasting blood triglycerides vary with circadian phase in both young and older people

**DOI:** 10.14814/phy2.14453

**Published:** 2020-06-09

**Authors:** Robin K. Yuan, Kirsi‐Marja Zitting, Wei Wang, Orfeu M. Buxton, Jonathan S. Williams, Jeanne F. Duffy, Charles A. Czeisler

**Affiliations:** ^1^ Division of Sleep and Circadian Disorders Departments of Medicine and Neurology Brigham and Women's Hospital Boston MA USA; ^2^ Division of Sleep Medicine Harvard Medical School Boston MA USA; ^3^ Department of Biobehavioral Health Pennsylvania State University University Park PA USA; ^4^ Division of Endocrinology, Diabetes, and Hypertension Department of Medicine Brigham and Women's Hospital and Harvard Medical School Boston MA USA

**Keywords:** aging, circadian, lipid panel, triglycerides

## Abstract

Daily rhythms in several physiological processes are important for cardiometabolic health. The lipid panel is used clinically to assess cardiovascular disease risk, but previous attempts to demonstrate circadian variation in lipids have failed to uncouple the endogenous circadian rhythm from the effects of meals and wake duration. Changes in basal lipid levels and dampening of circadian rhythms have been reported with aging, but it is unknown whether aging is also associated with changes in the rhythmic variation of lipids. We measured fasting lipid panels (triglycerides, total cholesterol, high‐density lipoprotein, and low‐density lipoprotein) in blood at wake time in 21 healthy adults using a specialized laboratory protocol that uncouples sleep–wake and activity‐related effects from the endogenous circadian rhythm. Young and older adults exhibited endogenous circadian variations in fasting triglycerides, with both groups peaking in the early biological morning. Young adults also exhibited significant circadian rhythmicity in total cholesterol and low‐density lipoprotein, while older adults did not exhibit circadian rhythmicity in any other lipids. These results reveal that triglyceride metabolism may be regulated by the central circadian pacemaker. Moreover, our findings may have clinical implications in assessing cardiovascular risk in shift workers and younger adults, since routine measurement of morning/fasting lipids may not fully and reliably assess triglyceride‐ and other lipid‐related biomarkers of cardiovascular disease risk in these groups.

## Graphical abstract

Daily rhythms in several physiological processes are important for cardiometabolic health. Young and older adults exhibited endogenous circadian variations in fasting triglycerides, with both groups peaking in the early biological morning. These results reveal that triglyceride metabolism may be regulated by the central circadian pacemaker, and may have clinical implications in assessing cardiovascular risk in shift workers and younger adults.

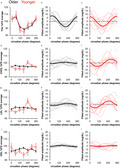

## 1 INTRODUCTION

Measurement of lipids in peripheral blood was one of the earliest biomarkers developed, and its use to assess both cardiovascular risk and the response to treatment is ubiquitous in clinical medicine. Daily rhythms in several physiological processes are important contributors to cardiometabolic health and cardiovascular risk (Crnko, Du Pre, Sluijter, & Van Laake, 2019; Morris, Yang, & Scheer, 2012; Scheer, Hilton, Mantzoros, & Shea, 2009). The disruption of these rhythms, as commonly seen in shift work, has been associated with adverse metabolic effects, including obesity, hypertension, impaired glucose regulation, and dyslipidemia (Antunes, Levandovski, Dantas, Caumo, & Hidalgo, 2010; Morris, Purvis, Mistretta, & Scheer, 2016).

Previous attempts to demonstrate diurnal or circadian variation in lipids have failed to uncouple the endogenous circadian rhythm from the effects of meals and wake duration (Ang et al., 2012; Chua et al., 2013; Dallmann, Viola, Tarokh, Cajochen, & Brown, 2012; Kasukawa et al., 2012; Poggiogalle, Jamshed, & Peterson, 2018). Moreover, although aging has been associated with changes in both cardiovascular disease risk and dampening of some circadian rhythms (Duffy, Zitting, & Chinoy, 2015), it is unknown whether aging is associated with changes in the rhythmic variation of lipids. These findings may have clinical implications for whether a single fasted lipid panel is sufficient for fully assessing triglyceride‐ and other lipid‐related biomarkers of cardiovascular disease risk.

We investigated the circadian influence on triglycerides (TGs), total cholesterol (CHOL), high‐density lipoprotein (HDL), and low‐density lipoprotein (LDL), independent of activity, sleep, and food intake in healthy young and older people using a specialized laboratory protocol, the forced desynchrony (FD) protocol.

## 2 METHODS

Data were collected in two FD protocols which have been described previously (Buxton et al., 2012; Zitting et al., 2018). The protocols were approved by the Partners Health Care Institutional Review Board (2014‐P‐000243, NCT02171273; 2005‐P‐002292), and adhered to the ethical principles outlined in the Declaration of Helsinki. All participants provided written informed consent. Exclusion criteria included current/chronic medical conditions; regular medication usage; BMI > 32; current/past psychiatric disorders; sleep disorders/complaints or excessive daytime sleepiness; abnormal values on a complete blood count, comprehensive metabolic panel, or routine urinalysis; smoking; and excessive caffeine/alcohol consumption. To ensure participants had stably entrained circadian rhythms, we excluded those who had done regular night shift work within the last 3 years, crossed more than two time zones within the last 3 months, or had habitual sleep durations <6.5 hr or >9 hr. All participants maintained regular sleep–wake schedules with overnight 10‐hr time‐in‐bed (TIB) for ≥3 weeks prior to admission.

Twenty‐one individuals (12 young: 7F, mean age 24 ± 5 yrs; 9 older: 6F, mean age 61 ± 5 yrs) completed the study (See Table 1 for participant characteristics). Three individuals completed the study twice under low‐ and high‐fat diet conditions, for a total of 24 separate trials. No individual completed the study more than once under the same conditions. Low‐fat diet consisted of 55%–60% carbohydrates, 15%–20% protein, and 20%–30% fat, and total calories for participant diets were calculated based upon either the Harris Benedict equation with an activity factor of 1.4 (Harris & Benedict, 1919) or the Mifflin‐St. Joer equation with an activity factor of 1.3 (Mifflin et al., 1990). High‐fat diet consisted of 30%–40% carbohydrates, 15%–20% protein, and 45%–50% fat, and total calories for participant diets were calculated based upon the Mifflin‐St. Joer equation with an activity factor of 1.6 (Mifflin et al., 1990). Both diets also included 150 mEQ Na+ (±20%), 100 mEq K+ (±20%), and at least 1.5 liters of fluid per 24 hr. Calories were distributed evenly across breakfast, lunch, and dinner in the low‐fat diet; during the FD segment, a snack consisting of 16.7% of the 24‐hr calorie target was served after dinner. In the high‐fat diet condition, two snacks consisting of 12.5% of the daily calories were served after lunch and dinner. In an effort to maintain stable participant weight throughout the study, the caloric content of the meals was adjusted when a participant's weight changed by more than 1 kg.

**TABLE 1:** Participant characteristics

Age years	Participant	Protocol	Diet	Sex	BMI kg/m^2^	TG		CHOL		LDL		HDL	
						Amplitude	Phase	Amplitude	Phase	Amplitude	Phase	Amplitude	Phase
						mean (SE)							
						mg/dL	degrees	mg/dL	degrees	mg/dL	degrees	mg/dL	degrees
18	2895	2005‐P‐002292	lower fat	f	20.9	6.2 (4.1)	359.4 (41.4)	4.7 (2.0)	246.1 (22.3)	5.3 (1.4)	241.5 (13.9)	0.8 (0.4)	148.1 (35.7)
18	3075	2005‐P‐002292	lower fat	f	22.4	3.1 (1.1)	117.6 (22.1)	6.0 (1.4)	236.0 (12.5)	11.3 (3.0)	211.7 (13.2)	6.6 (1.9)	5.3 (15.0)
20	28B2	2005‐P‐002292	lower fat	f	18.8	15.0 (7.7)	37.7 (30.4)	4.6 (9.1)	194.4 (127.5)	7.4 (8.3)	176.7 (73.5)	2.1 (0.9)	273.9 (20.9)
22	2701	2005‐P‐002292	lower fat	f	20.8	17.9 (6.4)	336.6 (21.7)	22.2 (12.9)	315.2 (37.2)	17.7 (9.6)	312.7 (34.8)	1.3 (2.7)	283.3 (125.6)
23	29U2	2005‐P‐002292	lower fat	m	20.3	10.8 (4.5)	316.0 (22.0)	7.0 (2.2)	278.0 (18.0)	7.1 (1.1)	220.9 (9.5)	4.9 (1.6)	353.1 (18.1)
24	30E1	2005‐P‐002292	lower fat	f	22.1	12.7 (3.0)	347.4 (15.0)	5.3 (3.5)	344.2 (41.8)	1.0 (2.3)	293.4 (110.7)	2.1 (1.5)	1.3 (51.0)
24	2903	2005‐P‐002292	lower fat	f	23.6	8.3 (2.2)	359.1 (16.8)	6.8 (1.9)	333.7 (17.0)	4.6 (1.6)	325.3 (20.6)	0.8 (0.3)	341.7 (26.7)
24	29D7	2005‐P‐002292	lower fat	f	29.4	6.5 (4.4)	55.1 (42.6)	4.4 (0.8)	319.2 (9.6)	6.2 (1.3)	304.1 (10.3)	1.2 (0.8)	123.6 (34.2)
25	3018	2005‐P‐002292	lower fat	m	22.8	6.4 (12.2)	172.7 (116.1)	8.0 (2.0)	280.1 (15.1)	14.4 (1.8)	268.8 (6.9)	6.7 (1.4)	63.0 (10.2)
26	3017	2005‐P‐002292	lower fat	m	23.0	7.8 (5.6)	311.6 (39.7)	16.5 (5.9)	233.7 (21.5)	12.2 (3.0)	220.4 (14.7)	4.2 (2.4)	251.5 (33.3)
27	3098	2005‐P‐002292	lower fat	m	20.8	12.0 (3.9)	8.0 (17.5)	9.2 (4.1)	250.2 (23.7)	5.1 (3.8)	237.2 (38.1)	2.2 (0.7)	275.0 (16.9)
38	2056	2014‐P‐000243	high‐fat	m	20.3	9.9 (2.4)	6.8 (13.0)	3.7 (1.9)	179.7 (27.0)	2.0 (1.7)	123.5 (59.1)	3.9 (0.4)	198.0 (4.5)
56	27D7	2005‐P‐002292	lower fat	f	23.5	4.2 (1.8)	352.1 (27.6)	3.5 (1.3)	76.6 (18.1)	2.9 (1.3)	82.1 (22.1)	0.8 (1.2)	110.1 (86.4)
56	3552*	2014‐P‐000243	lower fat	f	31.9	13.7 (3.6)	11.9 (14.7)	8.4 (2.5)	212.9 (15.9)	8.8 (1.8)	241.0 (11.2)	2.6 (0.9)	191.5 (19.2)
56	3552*	2014‐P‐000243	high‐fat	f	32.0	12.8 (4.0)	18.7 (18.4)	1.6 (1.4)	46.6 (46.3)	0.6 (1.1)	205.4 (102.9)	0.9 (0.4)	142.7 (27.2)
57	3536+	2014‐P‐000243	lower fat	f	30.6	13.8 (5.0)	63.2 (20.1)	0.9 (2.1)	302.5 (135.3)	1.6 (1.1)	275.0 (40.2)	1.9 (0.8)	245.1 (24.8)
59	3536+	2014‐P‐000243	high‐fat	f	30.9	5.0 (2.0)	34.5 (22.7)	2.3 (1.4)	158.4 (37.9)	3.0 (0.7)	168.7 (15.6)	0.5 (0.8)	260.9 (80.8)
59	2789	2005‐P‐002292	lower fat	f	21.3	10.0 (1.4)	18.6 (7.0)	5.9 (4.4)	158.9 (40.3)	7.8 (3.5)	185.1 (23.3)	2.0 (1.6)	74.8 (49.2)
60	29T2	2005‐P‐002292	lower fat	f	19.5	8.0 (2.1)	39.0 (12.9)	4.6 (3.8)	88.4 (38.5)	1.2 (2.3)	276.4 (96.4)	5.0 (2.9)	104.5 (31.0)
61	26P2	2014‐P‐000243	high‐fat	m	24.8	5.7 (3.0)	354.0 (29.9)	6.4 (2.8)	262.3 (25.5)	6.0 (2.9)	266.2 (27.8)	1.6 (0.8)	190.1 (27.3)
63	3453^	2014‐P‐000243	lower fat	m	30.1	6.1 (7.3)	34.4 (52.1)	3.7 (4.4)	58.4 (66.1)	2.3 (4.4)	29.7 (80.9)	1.8 (0.6)	131.1 (23.7)
65	3453^	2014‐P‐000243	high‐fat	m	30.0	10.2 (3.8)	17.1 (21.1)	3.5 (2.3)	178.6 (37.3)	2.4 (2.1)	188.1 (50.2)	3.0 (1.0)	185.2 (19.8)
69	3539	2014‐P‐000243	lower fat	f	19.9	7.9 (3.6)	23.2 (24.9)	2.9 (1.8)	298.1 (35.9)	2.2 (0.8)	258.7 (23.8)	1.2 (1.0)	291.8 (51.3)
70	28G2	2005‐P‐002292	lower fat	m	24.4	4.2 (2.2)	332.6 (36.4)	1.1 (1.2)	208.6 (50.6)	1.7 (0.8)	131.0 (32.9)	1.7 (0.9)	249.8 (26.9)

Note

Amplitude and acrophase for the individual fitted curve for each lipid are shown; bolded values indicate significance at the *p* < .05 level. Participants aged 18–38 yrs were classified as young adults. Participants 3552 (*), 3536 (+), and 3453 (^) each completed the study twice under different diet conditions.

All studies were carried out in the Intensive Physiological Monitoring Unit of the Center for Clinical Investigation at Brigham & Women's Hospital and began with six baseline/sleep extension days (8–16‐hr TIB). Participants then underwent 2–3 weeks of FD consisting of 28‐hr days (6 FD days per calendar week), with each day beginning 4 hr later than the previous day. Throughout FD, core body temperature (CBT) was recorded continuously with a rectal thermistor (Measurement Specialties, Inc., Dayton, OH) and edited to remove sensor slips and removals. Intrinsic circadian period of the CBT data from the FD segment of the protocol was estimated for each participant using non‐orthogonal spectral analysis (Czeisler et al., 1999). From this estimate, a circadian phase (from 0° to 359°) was assigned to each minute of the study, with 0° corresponding to the fitted CBT minimum.

Fasting blood samples were collected via an indwelling catheter within 40 min of wake time (<1 hr before breakfast) on 1–2 baseline days and on each 28‐hr day for 1 or 2 weeks during the FD segment. Samples were centrifuged and serum was sent for assay within 48 hr of collection (protocol 2014‐P‐000243), or plasma was stored at −80°C until assayed (protocol 2005‐P‐002292). Samples were assayed for CHOL, HDL, and TGs; LDL was estimated using the Friedewald formula (Lipid Panel, #303756; LabCorp).

Statistical analyses were carried out using SAS version 9.4 (SAS Institute). We tested for age differences by fitting a cosinor model at the group level with baseline as a covariate. We used linear mixed models to test for the effect of sex, study, diet, and BMI, none of which were significant and therefore not included in the final models. We included all available data from each participant as repeated measures in all statistical models. Repeat participants were included as nested random effects.

Lipids were considered rhythmic if both amplitude and phase were significant. To investigate whether loss of rhythmicity at the group level was due to amplitude dampening or phase dispersion, we fit cosinor models for each individual. Next, we ran cosinor models at the group level using the estimated peak and amplitude values from the individual fits (regardless of whether individual cosinor fits were significant), with peak phases aligned to 0° for all individuals. Data shown are mean ± *SD* unless otherwise specified. For figures, data were averaged within and across participants and binned into 60° (4 hr) circadian phase bins. Values are expressed as percentage of overall FD average in the figures. Only significant results (*p* < .05) are reported.

## 3 RESULTS AND DISCUSSION

**FIGURE 1:**
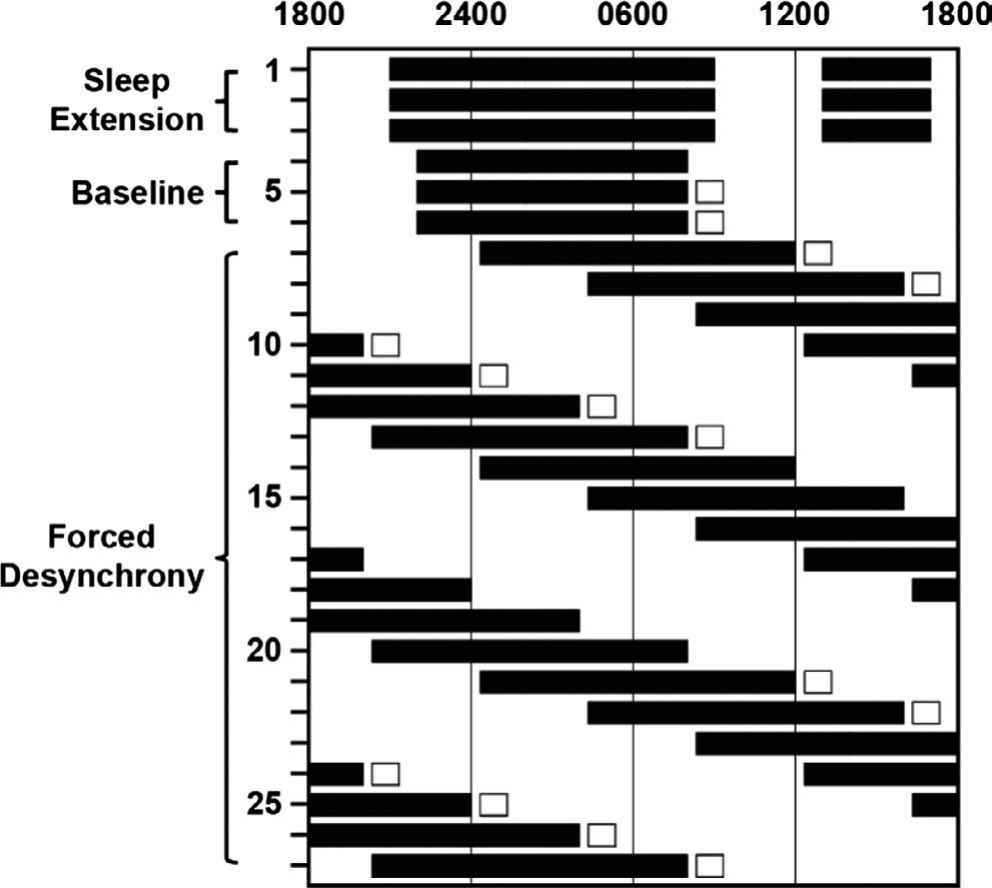
Schematic showing representative inpatient study schedule. Black bars indicate scheduled sleep opportunities, while white boxes indicate days that blood samples were drawn. All participants first underwent three sleep extension days with 16‐hr TIB and three baseline days with either 8‐ or 10‐hr TIB before beginning a 2–3 week FD segment. During FD, participants lived on 28‐hr “days” with 11.67 hr or 6.5 hr sleep opportunities. Fasting blood samples were drawn within 40 min of wake time during the first week, second week, or both first and third weeks of FD

We assessed fasting lipid levels in blood drawn across the full range of circadian phases using FD protocols in 24 trials conducted in 12 young and nine older participants (Figure 1). The FD protocol allowed us to separate sleep–wake, feeding, and activity‐dependent effects from the endogenous circadian rhythm of fasting lipid levels.

**FIGURE 2:**
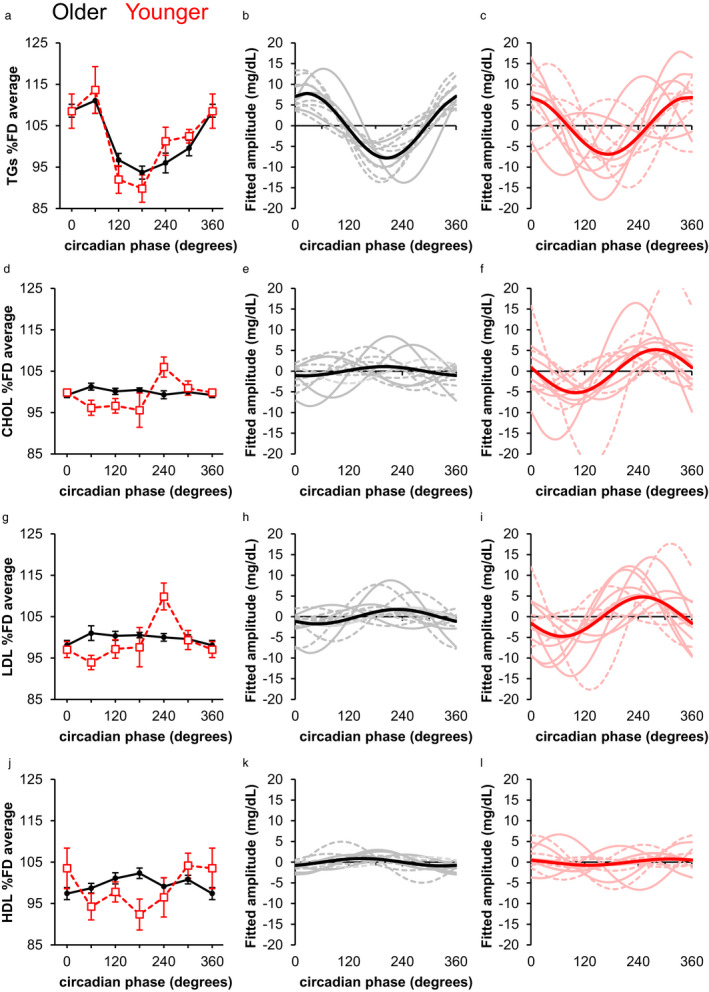
Circadian variation of fasting blood lipid levels. Left panels (a, d, g, j) show fasting blood lipid levels averaged with respect to circadian phase of the core temperature rhythm for younger (red) and older (black) adults. FD average ± *SD* in the young participants was 87.3 ± 55.5 mg/dl for triglycerides (a), 139.8 ± 38.0 mg/dl for total cholesterol (d); 92.8 ± 25.4 mg/dl for LDL (g), and 29.6 ± 9.7 mg/dl for HDL (j). FD average in the older participants was 95.8 ± 27.7 mg/dl for triglycerides (a), 172.3 ± 21.2 mg/dl for total cholesterol (d); 104.0 ± 17.0 mg/dl for LDL (g), and 49.1 ± 7.4 mg/dl for HDL (j). Middle panels (b, e, h, k) show individual (gray) and group (black) fitted curves in older adults, with dashed lines indicating individuals in whom we did not detect a significant circadian rhythm. Right panels (c, f, i, l) show individual (pink) and group (red) fitted curves in younger adults, with dashed lines indicating individuals in whom we did not detect a significant circadian rhythm. Values shown are means ± *SEM*

We found that young participants exhibited a significant circadian rhythm in fasting TGs (amplitude: *p* = .0002; phase: *p* < .0001; Figure 2a), consistent with previous results from non‐fasted and/or sleep‐deprived young adults (Chua et al., 2013; Dallmann et al., 2012; Kasukawa et al., 2012). Additionally, we report for the first time an endogenous circadian variation in TG levels in older adults (amplitude: *p* < .0001; phase: *p* = .0129; Figure 2a). These results, together with findings from studies using constant routine protocols that also report TG rhythms independent of meal timing (Chua et al., 2013; Dallmann et al., 2012; Kasukawa et al., 2012; Wehrens et al., 2017), suggest that TG metabolism may be regulated by the central circadian pacemaker. We observed a significant interaction of age and phase (*p* < .0001) but not age and amplitude in TGs, amounting to a difference of ~30° (2 hr) in the timing of the fitted peaks between young and older adults. After aligning the individual fitted TG peaks relative to the endogenous CBT nadir, there was no difference in amplitude between the two age groups, suggesting that there was no age‐related dampening of the TG circadian rhythm in these healthy older adults.

We observed greater individual variability in the timing of the fitted TG peaks relative to the temperature nadir in young (Figure 2b) compared to older (Figure 2c) participants. The mean distance from the individual fitted peaks to the group fitted peak in the young participants was 60.8 ± 44.4° (4 ± 3 hr). In contrast, in the older participants, the mean distance from the individual fitted peaks to the group fitted peak was 19.8 ± 16.5° (1.3 ± 1.1 hr), suggesting that there was less peak dispersion in the older participants. Similar interindividual differences in the timing of metabolite rhythms have been reported among younger adults (Ang et al., 2012; Chua et al., 2013; Dallmann et al., 2012; Kasukawa et al., 2012).

Elevated TG levels are associated with increased cardiovascular disease risk (Nordestgaard & Varbo, 2014). Field studies of healthy participants eating ad lib meals have shown that TG levels can exceed cardiovascular disease risk thresholds depending on the time of day when the sample is collected (Grant & Wolf, 2019). Moreover, non‐fasting TG levels vary with time of day even in carefully controlled laboratory studies in which food intake is evenly distributed throughout day and night in sleep‐deprived participants on a constant routine (Ang et al., 2012; Chua et al., 2013; Dallmann et al., 2012; Kasukawa et al., 2012; Wehrens et al., 2017). Although TG levels have been routinely assessed after an overnight fast, clinical guidelines in many countries now recommend the use of non‐fasting lipid panels for greater patient convenience (Langsted & Nordestgaard, 2019). These recommendations are based on data suggesting only limited differences between fasting and non‐fasting TG levels (Langsted & Nordestgaard, 2019), but may be confounded by time‐of‐day effects and interindividual differences, given that fasting TG levels exhibit circadian rhythmicity and substantial individual variability.

Young participants exhibited a significant rhythm in fasting total cholesterol (CHOL; amplitude: *p* = .0017; phase: *p* < .0001), while older participants did not (Figure 2d‐f). Aligning the peak phases revealed that older participants had significantly lower amplitudes for CHOL than younger participants (*p* = .0304). Similarly, young but not older participants exhibited a significant rhythm in fasting LDL (amplitude: *p* = .0008; phase: *p* < .0001; Figure 2g‐I). After aligning the fitted peaks, older participants had significantly lower amplitude for LDL than younger participants (*p* = .0120). Dampening of circadian rhythms has been associated with increased risk of metabolic disorders (Ando et al., 2009; Mantele et al., 2012; Sohail, Yu, Bennett, Buchman, & Lim, 2015), suggesting that robust rhythmicity may be important for metabolic health.

Mixed results have been reported regarding whether HDL concentrations vary with circadian phase (Poggiogalle et al., 2018). We found that neither older nor younger participants exhibited significant endogenous rhythms in fasting HDL at the group level (Figure 2j). Aligning the individual fitted peaks revealed no significant difference in amplitude between the age groups, but visual inspection revealed high individual variability in peak timing in younger participants (Figure 2l) compared to older participants (Figure 2k). The mean distance from the individual fitted peaks to the group fitted peak in the young participants was 105.5°±54.0° (~7.0 ± 3.6 hr), while the mean distance in older participants was 62.6°±41.1° (~4.2 ± 2.7 hr). Thus, some of the inconsistent findings previously reported in HDL rhythmicity are likely due to variability in peak timing between participants masking rhythmicity at the group level. Whether the dispersion in timing of the robust HDL rhythms among the young participants has implications for overall health remains unknown.

This study has some limitations. This is a post hoc analysis in which we combined data from two FD protocols conducted in highly controlled laboratory conditions collected over more than a decade, allowing us to accurately assess the relative lipid levels within individuals across the 24‐hr cycle while controlling for behavioral and environmental factors. However, to examine lipid levels independent of confounding medical conditions, we included only healthy young and older adults with no diagnosed medical, sleep, or psychiatric disorders who may not be representative of the general population. Moreover, individuals were studied under different dietary conditions, and although we found no effect of diet, our limited sample size does not allow us to rule out the possibility that diet may contribute to interindividual differences.

Overall, our study revealed that in healthy young and older adults, basal TG levels in blood collected after an overnight fast exhibit a robust endogenous circadian rhythm. Circadian rhythms in LDL and total cholesterol were dampened in older adults compared to young adults, while no significant circadian rhythm was detected in HDL in either age group. Further studies of this kind are needed in patients with cardiovascular disease to determine how such changes in the circadian rhythmicity of lipids impact health.

## CONFLICT OF INTERESTS

This study was supported by NIA P01 AG009975 and was conducted at the BWH Center for Clinical Investigation (CCI), part of Harvard Catalyst (Harvard Clinical and Translational Science Center) supported by NIH UL1 TR001102, BWH, and Harvard University and its affiliated academic health care centers. RKY was supported by T32HL007901 and F32HL143893. KMZ was supported in part by a fellowship from the Finnish Cultural Foundation. WW, JSW, and JFD report no conflict of interest. Outside the current work, OMB received subcontract grants to Penn State from Mobile Sleep Technologies (NSF/STTR #1622766, NIH/NIA SBIR R43AG056250), receives honoraria and travel support for Continuing Dental Education lectures at Tufts School of Dental Medicine, received honoraria and travel support for speaking at Boston College, Boston University and Allstate, received an honorarium for speaking for Allstate, and receives an honorarium for his role as Editor‐in‐chief (designate) of Sleep Health sleephealthjournal.org. CAC reports grants from Cephalon Inc., Jazz Pharmaceuticals Plc., Inc., National Football League Charities, Optum, Philips Respironics, Inc., Regeneron Pharmaceuticals, ResMed Foundation, San Francisco Bar Pilots, Sanofi S.A., Sanofi‐Aventis, Inc, Schneider Inc., Sepracor, Inc, Mary Ann & Stanley Snider via Combined Jewish Philanthropies, Sysco, Takeda Pharmaceuticals, Teva Pharmaceuticals Industries, Ltd., and Wake Up Narcolepsy; and personal fees from Bose Corporation, Boston Celtics, Boston Red Sox, Cephalon, Inc., Columbia River Bar Pilots, Ganésco Inc., Institute of Digital Media and Child Development, Klarman Family Foundation, Samsung Electronics, Quest Diagnostics, Inc., Teva Pharma Australia, Vanda Pharmaceuticals, Washington State Board of Pilotage Commissioners, Zurich Insurance Company, Ltd. In addition, CAC holds a number of process patents in the field of sleep/circadian rhythms (e.g., photic resetting of the human circadian pacemaker) and holds an equity interest in Vanda Pharmaceuticals, Inc. Since 1985, CAC has also served as an expert on various legal and technical cases related to sleep and/or circadian rhythms, including those involving the following commercial entities: Casper Sleep Inc., Comair/Delta Airlines, Complete General Construction Company, FedEx, Greyhound, HG Energy LLC, Purdue Pharma, LP, South Carolina Central Railroad Co., Steel Warehouse Inc., Stric‐Lan Companies LLC, Texas Premier Resource LLC, and United Parcel Service (UPS). CAC receives royalties from the New England Journal of Medicine; McGraw Hill; Houghton Mifflin Harcourt/Penguin; and Philips Respironics, Inc. for the Actiwatch‐2 and Actiwatch‐Spectrum devices. CAC’s interests were reviewed and managed by Brigham and Women's Hospital and Partners HealthCare in accordance with their conflict of interest policies.

## AUTHOR CONTRIBUTIONS

CAC, OMB, JFD, and JSW designed experiments. RKY and KMZ conducted experiments. RKY, KMZ, and WW analyzed data. RKY and KMZ prepared figures. RKY, KMZ, WW, OMB, JSW, JFD, and CAC wrote and reviewed the paper.
